# Biomaterials and biotechnology for periodontal tissue regeneration: Recent advances and perspectives

**DOI:** 10.34172/joddd.2022.001

**Published:** 2022-05-29

**Authors:** Rong Deng, Yuzheng Xie, Unman Chan, Tao Xu, Yue Huang

**Affiliations:** ^1^School of Stomatology, Jinan University, Guangdong, China; ^2^Department of Mechanical Engineering, Tsinghua University, Beijing, China

**Keywords:** Hydrogels, Periodontal regeneration, Three-dimensional printing, Tissue engineering

## Abstract

Periodontal tissues are organized in a complex three-dimensional (3D) architecture, including the alveolar bone, cementum, and a highly aligned periodontal ligament (PDL). Regeneration is difficult due to the complex structure of these tissues. Currently, materials are developing rapidly, among which synthetic polymers and hydrogels have extensive applications. Moreover, techniques have made a spurt of progress. By applying guided tissue regeneration (GTR) to hydrogels and cell sheets and using 3D printing, a scaffold with an elaborate biomimetic structure can be constructed to guide the orientation of fibers. The incorporation of cells and biotic factors improves regeneration. Nevertheless, the current studies lack long-term effect tracking, clinical research, and in-depth mechanistic research. In summary, periodontal tissue engineering still has considerable room for development. The development of materials and techniques and an in-depth study of the mechanism will provide an impetus for periodontal regeneration.

## Introduction

 Human tissue defects caused by diseases or traumas are challenges for medicine because human tissues have limited capabilities for regeneration and cannot meet the demand for in situ repair or ectopic tissue and organ transplantation.^1-3^ In recent years, with the progress of materials science and cellular and molecular biology, a brand new field, called tissue engineering and regenerative medicine, has developed as a promising strategy to alleviate the organ shortage crisis.^4,5^ Advanced manufacturing plays an essential role in this field.

**Figure 1:**
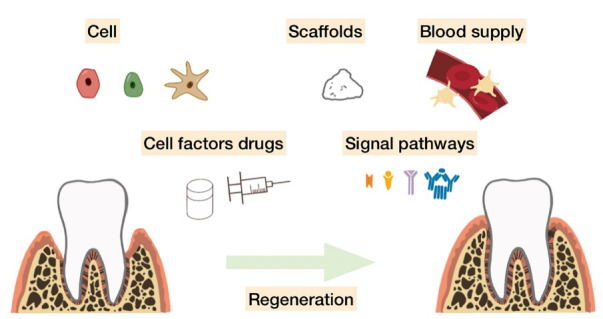
The factors involved in periodontal regeneration.

 Functional periodontal regeneration is a synergy of several factors (Figure 1). This review aims to highlight new frontiers in periodontal regeneration with a perspective on the application of biomaterials and emerging biotechnology. By summarizing their advantages and disadvantages, as well as existing possible solutions, this review provides references for future research directions.

## Physiological structure and regeneration forms of periodontal tissue

###  Physiological structure of periodontal tissue

**Figure 2:**
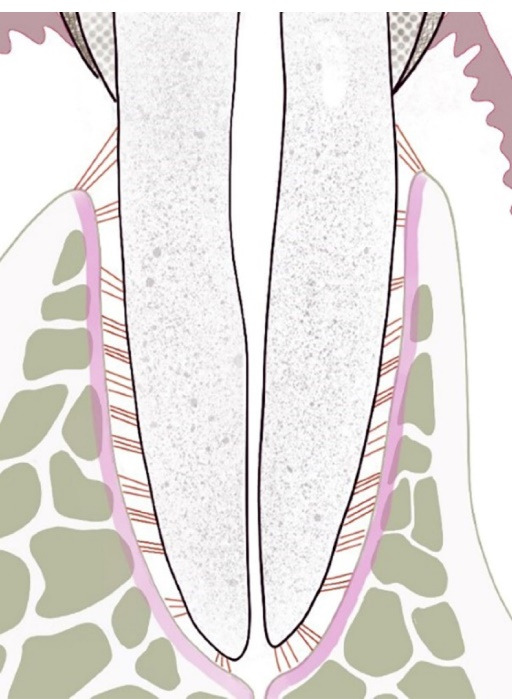
Schematic diagram of the basic structure of periodontal tissues.

 Periodontal tissue refers to a supporting tissue around teeth. It supports and fixes teeth in the alveolar socket and plays a decisive role in the retention and function of teeth. The components of periodontal tissue include the periodontal ligament (PDL), the cementum covering the surface of the tooth root, and the alveolar bone (Figure 2).^6^

####  Periodontal ligament

 The PDL, composed of connective tissue, is located in the space between the cementum and alveolar socket, with a thickness of 0.15–0.38 mm.^7,8^ It is composed of cells, matrix, fiber bundles, nerves, and blood vessels. PDL fiber bundles are synthesized by PDL fibroblasts, and both ends are embedded in the cementum and alveolar bone, which are called Sharpey’s fibers.^9^ The PDL has the functions of tooth retention, tooth nutrition, occlusal force dispersion, proprioception, sensory perception, and the ability to repair damaged periodontal tissue.^9,10^

####  Cementum

 In terms of anatomy, the cementum is a part of the tooth, but it is a part of the periodontium in terms of function. Its primary role is to provide attachment points for Sharpey’s fibers.^11^ Cementum is a thin and mineralized tissue covering the roots. There are two main structural forms: acellular cementum and cellular cementum. Acellular cementum is a calcified extracellular matrix with no cells. It is distributed on the surface of the dentin from the neck to the middle third of the root and is very important for attachment to the PDL. There are many depressions in the extracellular matrix of cellular cementum containing cementocytes. The cellular cementum mainly covers the root tip and plays a role in tooth movement and adaptation to the bite force after tooth germination.^12^ There is a layer of uncalcified cementum on the surface of the cementum, which is called cementoid. Through deposition, the cementum gradually thickens, forming a lamellar structure. The function of the cementum is to combine the periodontal tissues with teeth through Sharpey’s fibers and repair root surface damage through the deposition of cementum.^13^

####  Alveolar bone

 Alveolar bone is the socket in which the tooth roots are embedded in the upper and lower jaws, consisting of outer cortical plates of compact bone, a central substantia spongiosa, and bone lining the alveolus. The bone lining is where the PDL fiber bundles attach.^7^ The proper alveolar bone can be rebuilt due to stress. This function comes from the PDL.^14^

###  Forms of periodontal tissue regeneration

**Figure 3:**
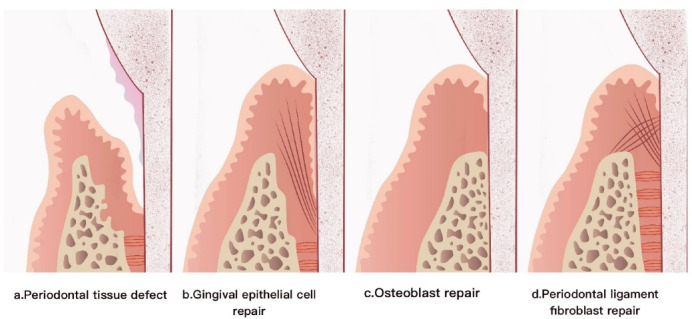
Three types of repair of periodontal defects.

 There are three main forms of periodontal tissue healing: long epithelial integration, PDL regeneration, and osseointegration (Figure 3).

####  Long epithelial integration

 Long epithelial binding is a stable form of healing. Compared with normal epithelium, it has lower cell proliferation ability.^15^ In the healing of periodontal tissues, oral epithelial cells will quickly form elongated junctional epithelium to facilitate organisms to resist bacterial infections. Epithelial cells quickly crawl and grow from the epithelium of the gingival surface to the wound surface, occupying the surface of the tooth root first until the surface of the tooth root is covered by thin and long combined epithelium, which affects the formation of new cementum on the surface of the tooth root and affects the integrity of periodontal attachment. This elongation of the combined epithelium usually leads to subgingival plaque formation and subsequent inflammation.

####  Periodontal ligament regeneration

 PDL regeneration is the ideal way of healing, which refers to the restoration of the lost PDL tissue to its original form and function. It involves the cooperation of two hard tissues (cementum and alveolar bone) and two soft tissues (gingiva and PDL).^16^ The surface of the tooth root exposed in the periodontal pocket forms new cementum, and at the same time, new alveolar bone is formed. When the two are regenerated, one end of the PDL fiber is buried in the cementum, and the other end is buried in the alveolar bone, forming new periodontal tissue.

####  Osseointegration

 Osseointegration, also known as bone ankylosis, is a process of the human immune system in which hard tissue is dissolved on the surface of the tooth root. When the tooth root is rebsorbed externally, and the damaged area is > 20%, bone remodeling is faster than the formation of cementum-like tissue; therefore, the cementum and dentin on the surface of the tooth root are rebsorbed by osteoclasts and replaced by bone tissue.^17^ The PDL is not formed, and the root and alveolar bone are closely connected.

## Materials for periodontal tissue regeneration

 Several materials are used as scaffolds for tissue engineering and regenerative medicine. The materials used for regenerating tissues must be biocompatible and biodegradable. Moreover, the degradation rate of the scaffolds should be consistent with the target tissue regeneration rate.^18^

 Polymers are common materials used for tissue engineering and regenerative medicine. They are classified as natural and synthetic polymers. Natural polymers are organic in origin and have good biocompatibility and biodegradability but insufficient mechanical properties. Synthetic polymers are produced industrially from inorganic sources and classified as absorbable and nonabsorbable polymers. Resorbable polyesters are predominant among synthetic polymers, including polycaprolactone (PCL), polylactic acid (PLA), polyglycolic acid (PGA), polylactic-polyglycolic acid (PLGA), polyethylene glycol (PEG), and PEG with PLGA (PEG-PLGA).^19^ Among them, the most representative synthetic polymer is PCL.

 PCL, an FDA-approved linear synthetic bioresorbable aliphatic polyester, has excellent thermal stability and is able to mold into different forms, which makes it different from the other materials used in scaffolds for tissue engineering.^20-22^ Moreover, it is hydrophobic and can hinder access to the medium and control drug dissolution.^23^ Unfortunately, due to its hydrophobicity, PCL is detrimental to cell attachment, proliferation, and differentiation.^24^ Hence, surface modifications are necessary. As PCL is easy to process, it has been used to repair various tissue defects via three-dimensional (3D) printing.

###  Materials for alveolar bone regeneration

 In the early days of periodontal tissue regeneration, researchers first focused on the regeneration of bone defects. Bone defects are the most important and obvious manifestation of periodontal defects and can be clearly observed in clinical x-ray examinations. Once the alveolar bone has rebsorbed, the tooth loses its bone support and loosens gradually, falling off eventually.

 Bioactivity, biocompatibility, and biodegradability are critical concerns in scaffold design, with important roles in bone regeneration.^25-28^ Moreover, the key parameters of porosity, stiffness, and viscoelasticity can regulate cell adhesion, proliferation, and osteogenesis differentiation.^29-36^ Well-designed scaffolds can provide cells with sustainable regenerative factors and physical and biological support, mobilizing stem cells to regenerate the defect cavity.^37-40^

 Collagen,^41^ chitosan,^42,43^ and gelatin^44^ are representative natural biomaterials with a favorable bone regenerative capacity because they share a similar extracellular matrix with the host and are suitable for cell migration, proliferation, and osteogenic differentiation. Interestingly, the in vivo metabolic components of these natural biomaterials are needed in the bone tissue reconstruction process.

 In addition, synthetic polymer-based biomaterials derived from a series of polymerization and crosslinking processes are designed purposefully with the expected properties and functions. Among them, PLGA and PCL^45^ with nontoxic, gelling, filming, and capsuling properties have found widespread applications.

 Calcium phosphate (CaP)-based bioceramics^46,47^ have found widespread applications, especially injectable CaP, with strong formability and flexibility. Nevertheless, the degradation of injectable CaP is limited, hindering the growth of new bones; therefore, it would be necessary to introduce porous materials with an enhanced degradation rate. Moreover, alloys are also a widely used bone repair material. The properties of natural or synthetic materials alone are slightly inferior, but when they are combined, a better bone repair result is realized.^48^

 A macroporous structure^49^ whose pores are > 100 μm allows angiogenesis and the migration of bone cells. It imitates the bone tissue structure and can significantly improve bone repair outcomes. Additionally, osteoinductive factors (e.g., bone morphogenetic protein-2 (BMP-2),^50^ fibroblast growth factor-2 (FGF-2), insulin growth factor (IGF), and platelet-derived growth factor-BB (PDGF-BB)) have essential roles in promoting osteogenesis.

###  Materials for periodontal ligament and cementum regeneration

 The PDL is the most important functional part of periodontal tissue, and its regeneration is of great significance. However, the PDL is a very thin layer of connective tissue between the alveolar bone and cementum. The morphology and structure are exquisite. Natural materials (such as collagen, chitosan, and gelatin)^51-53^and synthetic materials (such as PCL^54,55^ and PLGA^56^) are also suitable for PDLs. Moreover, because of their excellent fluidity and plasticity, hydrogel materials are very suitable for repairing PDL defects.

 Some researchers have optimized the properties of hydrogels by designing a green route to fabricate strong, supertough, regenerated cellulose films with tightly stacked and long-range aligned cellulose nanofibers. The study showed that this unique hierarchical structure could induce the adhesion and directional arrangement of cardiomyocytes, showing the potential for an oriented culture of cardiomyocytes in vitro. This advantage may be promising for inducing the directional arrangement of PDL fibers.^57-59^

 PCL can be electrospun^55,60^ and electrostatically written^61^ to produce PDL scaffolds. In particular, electrostatic direct writing technology, whose rotation direction is controllable, is very suitable for guiding the directional arrangement of PDL fibers.

 Few studies have fabricated scaffolds for the cementum because it occupies too little space. In studies that have produced a three-layer periodontal composite scaffold, the cementum layer is made of PCL/amelogenin^62^or chitin-poly (lactic-co-glycolic acid) (LGA)/nanobioactive glass ceramic (nBGC)/cementum protein-1.^63^ The addition of amelogenin and cementum protein 1 promotes cementum regeneration.

## Treatment for periodontal regeneration

###  Guided tissue regeneration

 The most commonly used clinical technology is guided tissue regeneration (GTR), a basic treatment applied to patients with periodontal defects. GTR uses membrane barriers that prevent epithelial cell proliferation and stimulate bone regeneration of the defect.^64^ GTR can be used only in some clinical cases, such as intraosseous defects and class II fissure defects. Several types of GTR membranes have been developed with improved physicochemical, mechanical, and biological properties to increase bone growth.^65,66^ However, GTR mainly promotes the repair of bone defects, while the regeneration of the PDL and cementum is still difficult to achieve.

###  Hydrogel

 Many agents and bioactive factors with strong anti-inflammatory, bone anabolic, and fiber anabolic effects have been studied preclinically, and their feasibility of periodontitis therapy has been confirmed. Hence, suitable drug delivery is necessary. Hydrogels are a good option because of their good fluidity, injectability, and biocompatibility.^67,68^ The emergence of photo-crosslinked hydrogels^69,70^ and thermosensitive hydrogels^67,71-73^ optimizes the performance of hydrogels as space-occupying scaffolds for periodontal defects. Hydrogels are crosslinked using a photoinitiator and a light lamp. The in situ thermoresponsive hydrogels maintain their fluidity at low temperatures, facilitating local injection through thin needles. Once administered in vivo, the solution solidifies into a hydrogel at body temperature, which would help maintain the drug payload for a long time in the periodontal pocket. In terms of biocompatibility and cell delivery, hydrogels also have excellent performance.^74-77^

###  Cell sheets

 Tissue engineering strategies based on cells and cell sheets have been widely used for periodontal tissue regeneration. Cell sheets, a strategy for seeding cell delivery to the periodontal defect area, have been introduced to regenerate periodontal tissues.^3,78^ The obtained cell sheet is placed between the root and alveolar bone. Human dental follicle cells and^79^ periodontal ligament stem cells (PDLSCs)^80-82^ have been used to seed the cells.

 The crosstalk of various cells is of great significance; for example, Zhang et al^78^ demonstrated that the crosstalk between PDLSCs and jaw bone marrow-derived mesenchymal stem cells in cell sheets facilitates the regeneration of complex periodontium-like structures. Yang et al^83^ demonstrated that human urine-derived stem cells promote the proliferation and osteogenic and cementogenic differentiation of PDLSCs in a ratio-dependent manner through noncontact coculture and further accelerate the regeneration of new structures by PDLSC sheets with osteogenic matrix in vivo. Safi et al^84^ isolated both PDLSCs and bone marrow mesenchymal stem cells and used them in a coculture method to induce more PDL cells to create three-layered cell sheets for reconstructing the natural PDL. By layering PDL cells and osteoblast-like cells on a temperature-responsive culture dish, Raju et al^85^fabricated a three-dimensional complex cell sheet composed of a bone-ligament structure. Ectopic and orthotopic transplantation results showed that the complex cell sheet group anatomically regenerated the bone-ligament structure along with the functional connection of PDL-like fibers to the tooth root and alveolar bone. Hence, coculture and crosstalk of cells provide a promising new strategy for the physiological and functional regeneration of periodontal tissue.

 Moreover, decellularized PDL cell sheets, which are a new technology, have also been confirmed to promote periodontal regeneration.^86^

###  3D printing

 3D printing is an emerging field, but there are few applications in periodontal tissue engineering, leaving a broad research space. 3D printing is a state-of-the-art additive manufacturing to turn 3D digital models into complex organs or other tissue constructs by fusing or depositing materials layer by layer through the head, nozzle, or another printer technology.^87^ Compared with the conventional tissue engineering methods, 3D printing has some apparent advantages. The conventional tissue engineering techniques cannot precisely control the pore size, geometry, and interconnectivity of the scaffolds; therefore, it is impossible to fabricate complex biomimetic tissue structures.^88^ Tissue constructs fabricated by conventional methods are too simplified to adapt to the native cellular microenvironment,^89^ while 3D printing is more likely to fabricate complex, precise, and individualized biomimetic tissue constructs with reproducibility and repeatability.^90-95^

 3D printing technology emerged in the 1980s. Charles Hull invented the world’s first 3D printer (stereolithography) in 1983.^96,97^ Since then, 3D printing techniques have made prompt advances. In 1987, selective laser sintering (SLS) was invented by Dr. Carl Deckard. In 1989, fusion deposition modeling (FDM) was devised by Scott Crump. Currently, in addition to SLS and FDM, the most popular 3D printing techniques are inkjet bioprinting, extrusion printing/bioprinting, and stereolithography.

 The 3D printing technique uses computer-assisted design and manufacturing after a CT scan. The scaffolds can be made of one or several materials, such as natural polymers, synthetic polymers, or both. They can be monophasic or multiphasic and tend to recreate the architectural structure of the periodontal tissue. Stem cells and/or growth factors can enhance bioactivity and promote regeneration.

 The ability to engineer bone-ligament interfaces is of significant interest for craniofacial systems.^98-101^ The integration of polarized fibers oriented to a mineralizing surface promotes adequate maturation and important biomechanical properties of the tissue, which regulates tissue adaptability and its long-term stability.^54^ Various approaches have been studied to encourage spatiotemporal control of multi-tissue formation and integration.^102,103^

 It is not easy to regenerate a single tissue, and periodontal regeneration involves three tissue types, which is even more difficult. Bone defects are the most obvious and often the largest defect; therefore, bone restoration is the first to attract researchers’ attention. The PDL, as an important functional component, carries out the functions of sensation, cushioning, nutrition, reconstruction, and restoration of alveolar bone and cementum, which cannot be ignored. As a result, with the development of science and technology, researchers began to produce biphasic scaffolds, including PDL cavities and alveolar bone cavities, to achieve the regeneration of alveolar bone and PDL fibers and form cementum.^55,56^ Another study found that adding a CAP coating to the alveolar bone scaffold can increase osteoconductivity and achieve more bone regeneration.^60^ Studies have also made a three-layer scaffold, including alveolar bone, PDL, and cementum, and added growth factors that can promote its regeneration in different parts to achieve the regeneration of three tissue types.^62^

 A very important issue in periodontal regeneration is the directional arrangement of PDL fibers, which is an essential factor in determining its function. Therefore, in some scaffolds, a structure to guide the arrangement of the fibers is designed. For example, in Park’s research, perpendicularly oriented channels were set to guide the direction of the fibers.^56^ Later, the team developed a fiber guiding scaffold to replace the previous random porous structure.^54^ A more favorable guiding fiber arrangement effect was realized.

 Park et al^104^ continued to conduct in-depth research on guiding the direction of PDL fibers. In 2014, they reported using directional freeze-casting techniques to control pore directional angulations and create topographies mimicking the alveolar crest and horizontal, oblique, and apical fibers of natural PDLs. Freeze casting is a simple approach that can create submicron-level porous constructs via aqueous materials^105,106^ because freezing conditions can control the microscopic patterns of ice crystals, and the regularity of ice growth can provide unidirectionally or radially oriented pores within the internal architectures.^107^ Other researchers on Park’s team have explored the effects of different depths and widths on grooved pillars for cell alignment, demonstrating increased cell alignment further from the pillar boundary in films with grooves compared to non-grooved pillars, with increased alignment in deeper-grooved (30 µm) pillars compared to shallow-grooved (15 µm) pillars.^108^ Moreover, a study added oriented nanofibers to the scaffold to guide the arrangement of new fibers.^109^ The electrostatic direct writing method is a new technology that is also effective in guiding the direction of the fiber.^61^

 Interestingly, scholars have extended the reconstruction of the PDL to implants. Cell sheets were applied around titanium implants. The results showed that cementum-like and PDL-like tissues were partly observed on the titanium surface.^110,111^

 In contrast with alveolar bone and PDL, the cementum has received less attention. On the one hand, the cementum is too thin, and the repair space is too small, which makes the production of 3D printing scaffolds very difficult. On the other hand, too few studies have focused on the mechanism of cementum formation. The cementum is secreted by cementoblasts. However, the regulatory mechanism of PDL stem cells or other stem cells differentiated into cementoblasts is not clear. Cementum formation may be related to the interaction between PDL cells and dentin.

 In 2015, Rasperini et al^112^ reported the first case of applying a 3D-printed scaffold to patients with clinical periodontal defects. Although this case was unsuccessful in the long term, it provided valuable experience for the clinical applications of periodontal regeneration. There was a large labial soft and osseous defect in the patient’s mandible. They designed and 3D-printed the scaffold using medical-grade PCL. Recombinant human platelet-derived growth factor-BB was delivered to the scaffold’s internal compartment. In the 13th month, the scaffold became exposed. Eventually, a larger dehiscence and wound failure were observed, and the entire scaffold was removed. The evaluation of the scaffold showed primarily connective tissue healing and minimal evidence of bone repair. The slow degradation rate of PCL might be the main cause of failure. The degradation rate of PCL did not match the rate of tissue formation, which caused the exposure of the scaffold and the invasion of bacteria. In addition, the structure of the scaffold was also of great significance. A highly porous structure inside the scaffold may promote the formation of blood vessels, which is beneficial to bone formation.

## Discussion

 Periodontal regeneration is significant to stomatology, but true periodontal regeneration is hard to achieve. The structure of periodontal tissue is very complicated. The sandwich structure of two kinds of hard tissues with a layer of soft tissue makes the construction of periodontal restoration difficult.

 At present, the rise of bioprinting provides a fresh impetus for periodontal regeneration. The continuous development of hydrogel materials will make them very promising periodontal restoration materials.^63,67,68,74,113-115^ The excellent biocompatibility, fluidity, plasticity, and injectability make hydrogels a suitable material to combine with bioprinting, particularly photo-crosslinked hydrogels and thermosensitive hydrogels.

 The weak mechanical properties of hydrogel materials can also be solved by adding cellulose.^58,59,116^ In addition, technology for the directional arrangement of cellulose in hydrogels has also been developed, which may greatly promote the development of technology for guiding the arrangement of PDL fibers.^57,117,118^

 However, the long-term effect of the method of guiding the orientation of the fibers is not clear. Moreover, the direction of the fibers may be inextricably linked to the surrounding force field. The adaptation of PDL cells to the bite force prompts them to continuously rebuild the PDL fibers and finally form a suitable arrangement direction.^119^

 Last but not least, the crucial point is the lack of understanding of the mechanism of periodontal tissue formation, especially cementum. In recent years, scholars have begun to study the mechanism of cementum regeneration, and the achievements are promising.^120^ CEMP1 and its peptide fragments have been confirmed to significantly affect cementum regeneration.^121-124^ Enamel-associated proteins and some other proteins have also been confirmed by related studies to promote cementum regeneration.^120^ Research on the mechanism and signaling pathways of cementum regeneration is still lacking, and there is broad room for research.

## Conclusion

 3D printing and bioprinting technology are promising technologies in periodontal regeneration. In the meantime, materials are developing by leaps and bounds. Only when researchers have a deeper understanding of the periodontal regeneration mechanism and technology continues to develop and improve can it be possible to apply their achievement to construct restorations and finally realize real periodontal regeneration.

## Authors’ Contribution

 RD, UC, and YX contributed to conceptualization, data curation, formal analysis, methodology, project administration, resources, software, writing an original draft, writing, review, and editing. YH and TX were conceptualization and supervision leads and finally read and revised the article.

## Funding

 This research received no external funding.

## Ethics Approval

 Not applicable.

## Competing Interests

 The authors declare no conflicts of interest.
